# Dataset of the first transcriptome assembly of the tree crop “yerba mate” (*Ilex paraguariensis*) and systematic characterization of protein coding genes

**DOI:** 10.1016/j.dib.2018.02.015

**Published:** 2018-02-10

**Authors:** Patricia M. Aguilera, Humberto J. Debat, Mauro Grabiele

**Affiliations:** a Instituto de Biología Subtropical (UNaM-CONICET) and Instituto de Biotecnología de Misiones, Universidad Nacional de Misiones, 3300 Posadas, Misiones, Argentina; b Instituto de Patología Vegetal, Centro de Investigaciones Agropecuarias (INTA), 5000 Córdoba, Argentina

## Abstract

This contribution contains data associated to the research article entitled “Exploring the genes of yerba mate (*Ilex paraguariensis* A. St.-Hil.) by NGS and *de novo* transcriptome assembly” (Debat et al., 2014) [1]. By means of a bioinformatic approach involving extensive NGS data analyses, we provide a resource encompassing the full transcriptome assembly of yerba mate, the first available reference for the *Ilex* L. genus. This dataset (Supplementary files 1 and 2) consolidates the transcriptome-wide assembled sequences of *I. paraguariensis* with further comprehensive annotation of the protein coding genes of yerba mate via the integration of *Arabidopsis thaliana* databases. The generated data is pivotal for the characterization of agronomical relevant genes in the tree crop yerba mate -a non-model species- and related taxa in *Ilex*. The raw sequencing data dissected here is available at DDBJ/ENA/GenBank (NCBI Resource Coordinators, 2016) [2] Sequence Read Archive (SRA) under the accession SRP043293 and the assembled sequences have been deposited at the Transcriptome Shotgun Assembly Sequence Database (TSA) under the accession GFHV00000000.

**Specifications Table**

**Table:** 

Subject area	Agronomy-Biology
More specific subject area	Transcriptomics
Type of data	Assembly of reads and sequence annotation
How data was acquired	Bioinformatics approach
Data format	Analyzed
Experimental factors	RNA, used for library construction and Illumina sequencing, was isolated from leaf samples at emerging, young, fully expanded, and early and late senescent stages from *I. paraguariensis* breeding line Pg538
Experimental features	Paired-end 100 nt Raw reads were filtered and *de novo* assembled. Transcripts were submitted to in-house batch BlastX/tBlastn searches [3] using *Arabidopsis* as reference to characterize the protein coding genes of yerba mate and to find out putative orthologous genes between the two species
Data source location	Misiones, Argentina
Data accessibility	Data are within this article and at DDBJ/ENA/GenBank under the accessions SRP043293 and GFHV00000000

**Value of the data**

• This data provides full transcriptome assembled sequences of yerba mate, the first references for the *Ilex* L. genus.
• Data is applicable for the characterization of agronomical important genes in yerba mate and related taxa in *Ilex*.
• Accessibility of assembly and annotation data allows scientific community to implement additional analysis via original approaches.

## 1 Data

The data shared with this data article comprise Supplementary files 1 and 2. Supplementary file 1 presents transcriptome-wide assembled sequences of *Ilex paraguariensis* SRA SRP043293 (FASTA). Supplementary file 2 refers to the annotation of these assembled sequences via the integration of *Arabidopsis thaliana* protein databases (spreadsheets format).

## 2 Experimental design, materials and methods

Total RNA extracted of five samples of emerging, young, fully expanded, and early and late senescent stages leaves of *I. paraguariensis* breeding line Pg538 were pooled for high throughput sequencing.

The complete raw sequencing data at SRA under the accession SRP043293 [1], [2] was used to generate a full transcriptome assembly employing the Trinity 2.0.6 platform [4]. All raw sequenced reads were quality filtered and then *de novo* assembled -using optimal parameters of 25 kmer word and group pairs distance of 500- into 44,840 transcripts (~ 180X coverage) which encompass ca. 31,694 genes and their respective isoforms (13,146) in agreement to the Trinity output (Supplementary File 1, FASTA).

For the first step of annotation analysis, the whole genome information of the model species *A. thaliana* L. (TAIR10; http://www.arabidopsis.org) was downloaded into a local server system. Subsequently, the complete yerba mate translated transcriptome (269,040 sequences) was scanned by in-house [3; v.11.0.2] batch homology searches via BlastX (matrix Blosum62, word size 3, cut off value of 1e−05) using as bait the TAIR10 proteome (35,386 peptides of 27,416 gene models). In addition, tBlastn searches (matrix Blosum62, word size 3, cut off value of 1e-05) were performed using TAIR10 proteome as query and the complete yerba mate translated transcriptome as target. For both, direct and reverse searches, the best hit strategy was applied.

Both BlastX and tBlastn searches results were organized in different spreadsheets (Supplementary File 2, sheet 1 and 2, respectively) integrating several indicators, i.e. query name, subject name, e-value, bit-score, % query coverage, % pairwise identity, cumulated total alignment length, frame.

**Fig. 1:**
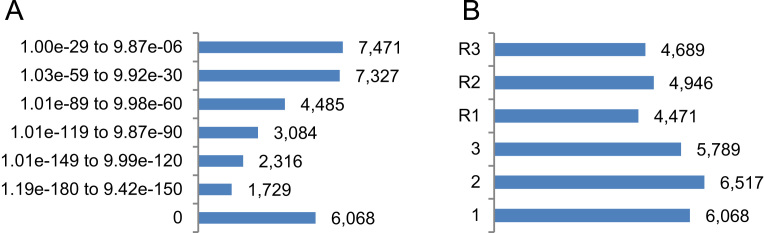
32,480 BlastX annotated transcripts classified according to e-value ranges (A) and direct and reverse (R) frames (B).

BlastX revealed 32,480 hits out of 44,840 transcripts of yerba mate (72.4%), embracing 21,370 genes (out of 31,694; 67.4%) and 11,110 isoforms (out of 13,146; 84.5%) which targeted 12,435 gene models of *Arabidopsis* (out of 27,416; 45.3%). Complete BlastX results displayed the following mean parameters: e-value of 4.42e−08, bit-score of 329.5, % query coverage of 73.7, % pairwise identity of 64.7 and cumulated total alignment length of 258.2 nucleotides. Mean parameters for each category considering e-value ranges are shown in Table 1. Roughly 40 % of the annotated transcripts (0 ≤ e ≤ − 90; Fig. 1A) exceed those mean parameters. Most yerba mate transcripts were annotated according to direct frames (56.6 %; Fig. 1B).

**Table 1:** 32,480 BlastX annotated transcripts classified according to e-value ranges and mean parameters.

**# YM transcripts**	**e-value**	**Bit-score**	**% query coverage**	**% pairwise identity**	**Alignment length**
6,068	0	874.7	80.5	71.5	625.1
1,729	1.19e−180 to 9.42e−150	476.1	76.0	68.6	366.4
2,316	1.01e−149 to 9.99e−120	393.8	74.5	68.1	308.6
3,084	1.01e−119 to 9.87e−90	314.8	73.9	66.8	261.3
4,485	1.01e−89 to 9.98e−60	230.8	74.4	66.4	193.0
7,327	1.03e−59 to 9.92e−30	148.0	76.8	65.0	128.7
7,471	1.00e−29 to 9.87e−06	76.1	63.8	55.0	84.5

**Fig. 2:**
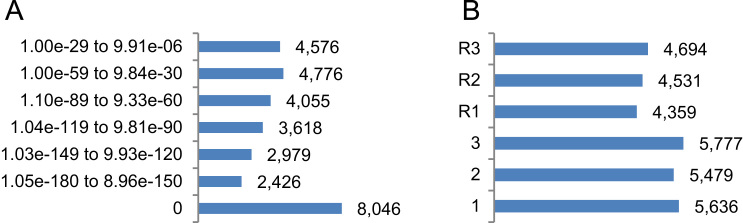
30,476 tBlastn annotated transcripts classified according to e-value ranges (A) and direct and reverse frames (B).

In addition, through tBlastn approach, 30,476 sequences of *A. thaliana* proteome database (out of 35,386; 86.1%), embracing 23,033 gene models (out of 27,416; 84.0%), found yerba mate hits. Those hits belong to 10,904 unique transcripts − 24.3 % of yerba mate transcriptome- of 9,885 genes (out of 31,694; 31.2%). Complete tBlastn results displayed the following mean parameters: e-value of 3.09e−08, bit-score of 404.9, % query coverage of 79.5, % pairwise identity of 59.0 and cumulated total alignment length of 351.7 nucleotides. Mean parameters for each category considering e-value ranges are shown in Table 2. Near 46% of the annotated transcripts (0 ≤ e ≤ − 120; Fig. 2A) are above those mean parameters. Most yerba mate transcripts were annotated according to direct frames (55.4%; Fig. 2B).

**Table 2:** 30,476 tBlastn annotated transcripts classified according to e-value ranges and mean parameters.

**# YM transcripts**	**e-value**	**Bit-score**	**% query coverage**	**% pairwise identity**	**Alignment length**
8,046	0	851.6	93.2	69.7	623.2
2,426	1.05e−180 to 8.96e−150	475.0	89.8	63.3	412.3
2,979	1.03e−149 to 9.93e−120	393.4	86.3	62.1	355.1
3,618	1.04e−119 to 9.81e−90	314.7	82.7	59.4	311.0
4,055	1.10e−89 to 9.33e−60	232.7	77.3	58.1	246.2
4,776	1.00e−59 to 9.84e−30	152.1	69.1	52.6	190.7
4,576	1.00e−29 to 9.91e−06	77.8	55.7	43.3	133.7

Finally, BlastX and tBlastn results were merged, curated and organized according to consecutive gene model names of *Arabidopsis* (Supplementary File 2, sheet 3) in order to detect reciprocal best hits (RBH) and vague results amongst both search approaches, in addition to putative gene duplications. Complete merged list embrace 62,956 yerba mate transcripts, from which 32,498 are unique and include 21,387 genes and 11,111 isoforms, currently linked to 23,052 protein gene models of *Arabidopsis*.

RBH strategy is useful to infer orthologous relationships among protein gene datasets [5]. However, to finally decide on the orthology of pair-wise aligned sequences, additional criteria should be considered, i.e. e-value, bit-score, % pairwise identity, cumulated total alignment length, visual inspection of the alignment [6]. Our analysis in yerba mate revealed 9,437 BlastX/tBlastn RBH pairs *sensu stricto* (equivalent *Arabidopsis* gene model peptide/yerba mate gene isoform), including 9,244 gene pairs out of 21,387 annotated genes (43.2%). From those, 4,764 gene pairs and their respective 10,683 unique isoforms can be grouped as RBH *sensu lato* (equivalent *Arabidopsis* gene model/yerba mate gene). Another 437 yerba mate genes, unrelated to the RBH *sensu stricto* group, and their respectives 1,292 unique isoforms can be grouped as RBH *sensu lato* also (see Supplementary File 2, sheet 3). RBH *sensu stricto* annotated pairs displayed the following mean parameters: e-value of 7.25e−09, bit-score of 485.9, % query coverage of 78.7, % pairwise identity of 66.6 and cumulated total alignment length of 368.0 nucleotides. Mean parameters for each category considering e-value ranges are shown in Table 3. Around 44% of the RBH *sensu stricto* annotated transcripts (0 ≤ e ≤ − 150) are above those mean parameters.

**Table 3:** 9,437 RBH *sensu stricto* annotated pairs classified according to e-value ranges and mean parameters.

**# YM transcripts**	**e-value**	**Bit-score**	**% query coverage**	**% pairwise identity**	**Alignment length**
6,748	0	876.1	87.1	71.5	623.0
1,563	1.16e−180 to 9.42e−150	472.1	82.7	68.1	366.0
1,933	1.01e−149 to 9.93e−120	389.1	80.6	67.7	306.0
2,280	1.01e−119 to 9.74e−90	310.2	77.5	65.9	258.0
2,541	1.03e−89 to 9.98e−60	228.4	73.8	64.5	199.0
2,525	1.00e−59 to 9.92e−30	150.0	68.4	61.6	143.0
1,284	1.03e−29 to 9.91e−06	80.4	59.2	52.7	99.0

In sum, we performed an integrated high-throughput screening analyses, based in BlastX/tBlastn strategy and employing highly curated databases of *A. thaliana*, the most extensively studied plant. Our approach resulted in a comprehensive annotation of over 21,387 yerba mate genes and prediction of 9,874 orthologous genes among both species.

This Transcriptome Shotgun Assembly project has been deposited at DDBJ/ENA/GenBank under the accession GFHV00000000. The version described in this paper is the second version, GFHV02000000.
